# Single-layer metasurface for ultra-wideband polarization conversion: bandwidth extension via Fano resonance

**DOI:** 10.1038/s41598-020-79945-0

**Published:** 2021-01-12

**Authors:** Zhongtao Zhang, Jiafu Wang, Xinmin Fu, Yuxiang Jia, Hongya Chen, Mingde Feng, Ruichao Zhu, Shaobo Qu

**Affiliations:** Department of Basic Science, Air Force Engineering University, Xi’an, 710051 Shanxi People’s Republic of China

**Keywords:** Materials science, Physics

## Abstract

In this paper, we propose a method of designing ultra-wideband single-layer metasurfaces for cross-polarization conversion, via the introduction of Fano resonances. By adding sub-branches onto the unit cell structure, the induced surface currents are disturbed, leading to coexistence of both bright and dark modes at higher frequencies. Due to the strong interaction between the two modes, Fano resonance can be produced. In this way, five resonances in all are produced by the single-layer metasurface. The first four are conventional and are generated by electric and magnetic resonances, whereas the fifth one is caused by Fano resonance, which further extends the bandwidth. A prototype was designed, fabricated and measured to verify this method. Both the simulated and measured results show that a 1:4.4 bandwidth can be achieved for both *x-* and *y*-polarized waves, with almost all polarization conversion ratio (PCR) above 90%. This method provides an effective alternative to metasurface bandwidth extension and can also be extended to higher bands such as THz and infrared frequencies.

## Introduction

Metamaterials refer to artificial objects exhibiting intriguing properties which do not exist in nature^1–4^. Metamaterials can achieve a mass of exotic physical effects, such as polarization conversion^5–7^, negative refraction^8^, perfect absorber^9,10^ and so on. In order to reduce bulky volume and be more suitable for application, a two-dimensional form of metamaterial is generated, called as metasurface, which is a planar array composed of sub-wavelength metamaterial units. In the past decade, study of polarization converter is quite familiar to scientific community, but it is still a key issue in electromagnetic (EM) wave. Many meaningful and excellent works have been proposed such as linear-to-linear, linear-to-circular and dual-band polarization^11–15^. If a facile structure can achieve more wideband and higher-efficiency simultaneously, it will further promote the development of various polarization manipulating devices such as high-efficient circularly polarized antennas^16,17^, more reduction of radar cross section (RCS)^18,19^, and even to construct more complex EM waves^20^.

Many polarization converters based on anisotropic metasurfaces^21,22^ and chiral metasurfaces^23,24^ have been accounted. However, these polarizers suffer from either the narrow bandwidth or the low-efficiency, which extremely limit their practical applications. One method to extend the bandwidth is to stack multilayers^25^, which will lead to bulky thickness and difficult preparation. The other method is to use multiple resonances. Chen et al*.*^6^ have indicated that an ultra-wideband metasurface can achieve a 1/4 3 dB bandwidth through four electric and magnetic Lorentzian resonances but the polarization conversion ratio (PCR) is only above 50%. Therefore, it is a priority for us to expand bandwidth under the condition of maintaining the high-efficiency.

Fano resonances have been research hotspots recently, due to their capability of providing ultra-narrow spectral linewidth and hence various potential applications such as sensing, slow light, and nano-lasing^26–28^. Fano resonances is viewed as a quantum interference between a discrete state and a continuum state in atomic physics^23,29,30^. The shape of Fano resonance is distinctively different from the most common observed spectral line shapes known as symmetric Lorentzian line shapes. It is characterized by an asymmetric spectral shape and have been achieved in various electromagnetic metamaterial structures^31^.

**Figure 1:**
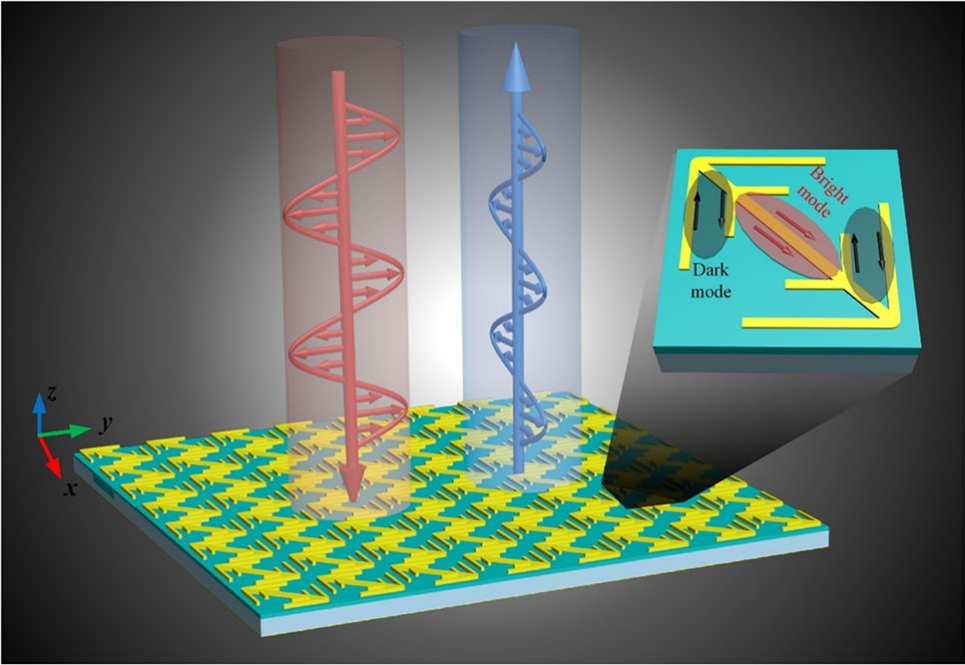
Schematic of polarization conversion using Fano resonance formed by interaction between dark mode and bright mode.

At present, most wideband polarization rotators are created by increasing the number of Lorentzian resonances but seldom involve Fano resonances^5,6,18,19,32,33^. In this paper, we aim to further extend the bandwidth via the introduction of Fano resonance. To this end, we first give an optimized double-head arrow (hereafter called unit 1) that consist of one long straight cut-wire and two oblique V-shaped wires, according to multiple plasmon resonances of Chen et al*.*^6^. Next, two sub-branches are added to the unit 1, generating a composited structure (hereafter called unit 2). When combine them together, the canceling current will be formed between the branch and sub-branch, which can be regarded as dark mode, as shown in Fig. 1. Due to the strong interaction between the bright and dark modes, Fano resonance can be produced. Ultimately, after optimization design, a single-layer ultra-wideband linear polarization conversion is proposed. It is demonstrated that five resonances in all are produced by the single-layer metasurface. The first four are conventional and are generated by electric and magnetic resonances, whereas the fifth one is caused by Fano resonance, which further extends the bandwidth for cross-polarization reflection. Both the simulated and measured results show that a 1:4.4 bandwidth can be achieved for both *x*- and *y*-polarized waves, with almost all polarization conversion ratio (PCR) above 90%.

## Theory design

**Figure 2:**
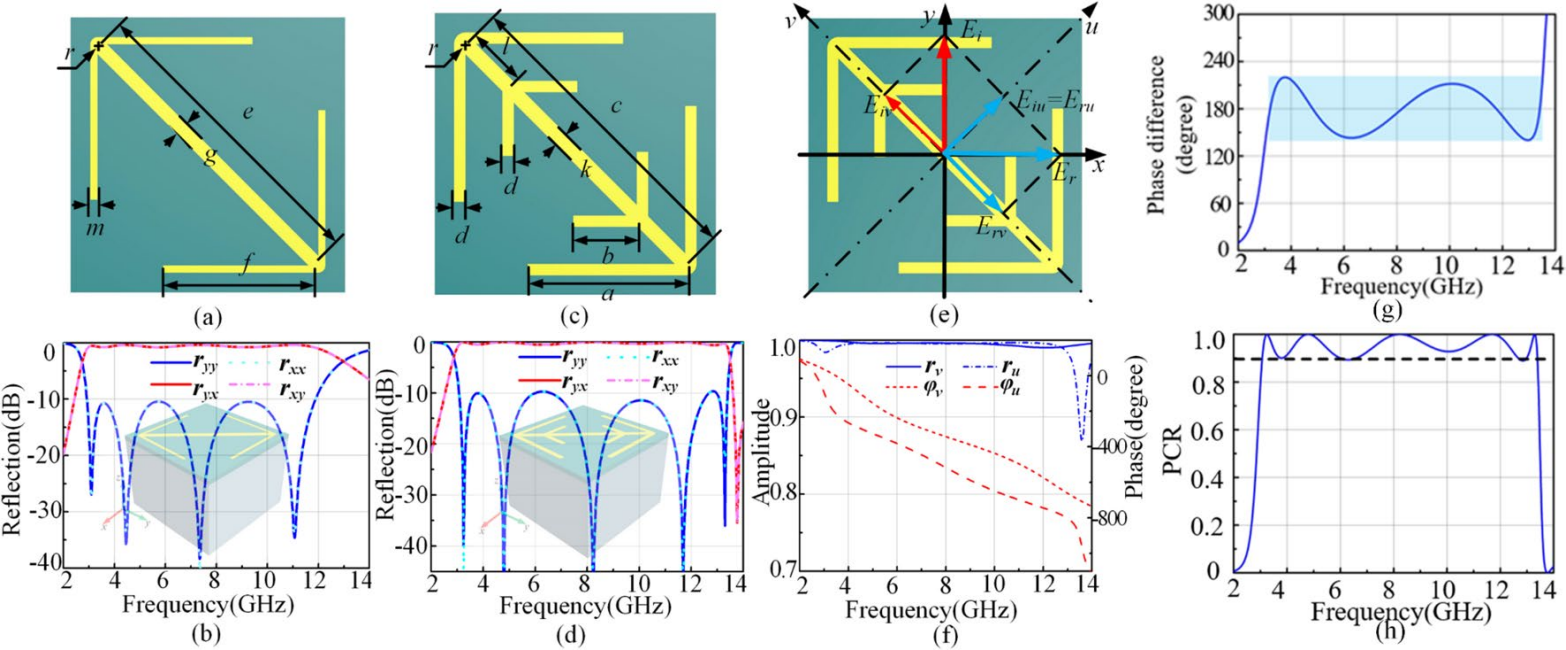
Top view **(a)** and simulated co- and cross-polarization reflections **(b)** for unit 1. Top view **(c)** and simulated co- and cross-polarization reflections **(d)** for unit 2. **(e)** is the intuitive image of *y*-polarized incident wave rotated to *x*-polarized reflection wave. **(f)** The reflection amplitudes and phases of unit 2 for *u*- and *v*-polarized waves. **(g)** The phase difference between *u*- and *v*-polarized waves. **(h)** The simulated PCR versus frequency under normal incidence.

Chen et al.^6^ have indicated that the bandwidth can be broadened through multiple plasmon resonances. On the basis of it, we give the unit 1 shown in Fig. 2a. The periodicity of unit 1 is 12.2 mm. Unit 1 can be divided into four layers. The thickness of the top copper pattern and background sheet is 0.018 mm with a conductivity of $5.8\times {10}^{7}$S/m. There are 0.5 mm F4B dielectric layer (a dielectric constant 2.65 and a loss tangent 0.001) and 8.0 mm foam layer (a dielectric constant 1) in the middle of them. The other parameters are *e* = 13.7 mm, *f* = 6.95 mm, *g* = 0.70 mm, *m* = 0.30 mm, *r* = 0.5 mm. Then, full-wave simulations are performed using the frequency domain solver in CST Microwave Studio with periodic boundary conditions in *x*- and *y*-directions and open conditions along the *z*-direction. The polarization conversion properties are studied by the reflection coefficients. ${r}_{yy}$(${r}_{xx}$) is the co-polarization reflection coefficient and ${r}_{xy}$(${r}_{yx}$) is the cross-polarization reflection coefficient under *y*- (*x*-) polarized wave. As shown in Fig. 2b, unit 1 has a 10 dB bandwidth from 2.91 GHz to 11.88 Hz with four Lorentzian resonances.

On the other hand, hoping to get Fano resonance to broaden bandwidth of polarization conversion, two sub-branches are added to the unit 1, generating the unit 2 depicted in Fig. 2c. The unit 2 is periodically arranged along the *x*- and *y*-directions with a periodicity of *p* = 12.2 mm. Top layer is a 0.018 mm metal pattern, whose geometrical parameters are illustrated as follows: *a* = 6.95 mm, *b* = 2.82 mm, *c* = 13.7 mm, *d* = 0.5 mm, *k* = 0.7 mm, *l* = 2.51 mm, *r* = 0.5 mm. The second layer is F4B dielectric spacer with a thickness ${h}_{1}$=0.5 mm. The third layer is foam substrate with a thickness ${h}_{2}$=7.6 mm. And the bottom layer is a 0.018 mm copper ground sheet. Figure 2d shows the simulated cross-polarized reflection coefficients is more than − 1 dB from 3.01 GHz to 13.40 GHz for both *y*-polarized and *x*-polarized incident waves. Compared with Fig. 2b, we find that unit 2 does have one more resonance after adding two sub-branches and the bandwidth is further expanded, indeed.

The PCR is used to evaluate the performance of the polarization conversion, which is defined as $PCR={r}_{yx}^{2}/{(r}_{yx}^{2}+{r}_{xx}^{2})$. Therefore, we calculate the simulated PCR shown in Fig. 2h and find that almost all PCR is above 90% in the frequency range from 3.05 GHz to 13.40 GHz (about 1:4.4 bandwidth). The PCR can reach 100% in the frequency of 3.25 GHz, 4.81 GHz, 8.24 GHz, 11.69 GHz, and 13.30 GHz, respectively.

To better understand the principle of polarization conversion, *u*- and *v*-coordinate system is introduced. The *v*-axis is along 45° direction with respect to the *y*-axis and *u*-axis is perpendicular to *v*-axis, as shown in Fig. 2e. Then, we consider that the incident EM wave is polarized along the *y*-axis. The electric field can be decomposed into two orthogonal components (directions *u* and *v*). Hence, the electric field of the incident EM wave can be expressed as $\overrightarrow{\mathrm{E}}=\overrightarrow{u}{\mathrm{E}}_{iu}{e}^{j\varphi }+\overrightarrow{v}{E}_{iv}{e}^{j\varphi }$, where $\overrightarrow{u}$ and $\overrightarrow{v}$ are the unit vectors in the *u*- and *v*-axes, respectively. The electric field of the reflected wave can be written as $\overrightarrow{{E}_{r}}={r}_{u}\overrightarrow{u}{\mathrm{E}}_{iu}{e}^{j\varphi }+{r}_{v}\overrightarrow{v}{E}_{iv}{e}^{j\varphi }$, in which ${r}_{u}$ and ${r}_{v}$ are the reflection coefficients along the *u*- and *v*-axes, respectively. Due to the anisotropic characteristic of the metasurface, a different phase change $\mathrm{\Delta \varphi }$ can be generated. Therefore, when $\mathrm{\Delta \varphi }\approx\uppi$ and amplitude satisfy ${\mathrm{r}}_{\mathrm{u}}\approx {\mathrm{r}}_{\mathrm{v}}$, the field synthesized by ${E}_{ru}$ and ${E}_{rv}$ will be along the *x*-direction. The incident polarization is rotated by 90°. To verify the polarization conversion performance of the unit 2, we carried out numerical simulations of reflection phase and amplitude characteristics. As displayed in Fig. 2(f), the reflection amplitudes of *u*- and *v*-polarized waves are basically equal and we get 180°−39°$\le \mathrm{\Delta \varphi }\le$ 180° + 39° in Fig. 2(g) from 3.01 GHz to 13.40 GHz. Moreover, the minimum value of ${r}_{yy}$ almost appears at the frequency where $\mathrm{\Delta \varphi }$ is equal to 180°, further proving the correctness of the proposed criterion.

## Simulated results analysis

**Figure 3:**
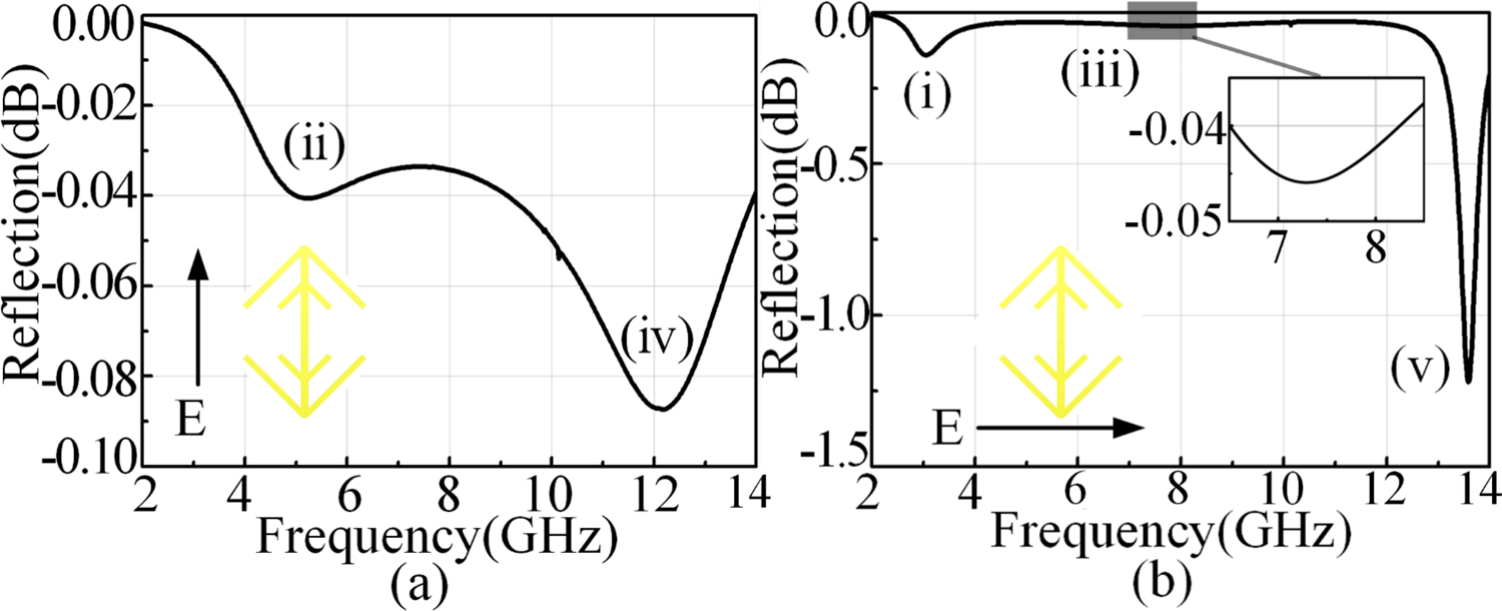
The five eigen-modes of unit 2 co-polarization reflections under normal incidence: *v*-polarized case **(a)** and *u*-polarized case **(b)**.

The curve of Fano resonance is asymmetry, which is a typical feature different from that of conventional symmetric resonance curves. As shown in Fig. 2d, the fifth resonance is asymmetry because of the dramatically decrease at the fifth resonance. Therefore, we can predict that unit 2 has a Fano resonance. In order to validate this, we study the resonance eigen-modes of unit 2 using CST Microwave Studio. The electric field component along *u*- and *v*-axes can excite resonant eigen-modes. Figure 3a,b present the simulated results, indicating that there are five dips. The three eigen-modes (i), (iii) and (v) are excited by *u*-polarized EM waves while the other two eigen-modes (ii) and (iv) are excited by *v*-polarized EM waves. Thus, when the *y*-polarized waves are incident, five resonances can be excited simultaneously because the *y*-polarized waves have both *u*- and *v*-components. However, there is a special resonance, the eigen-mode (v). We find that co-polarization reflection in (v) is far less than others. It means the resonance in (v) is fundamentally different from the other resonances.

**Figure 4:**
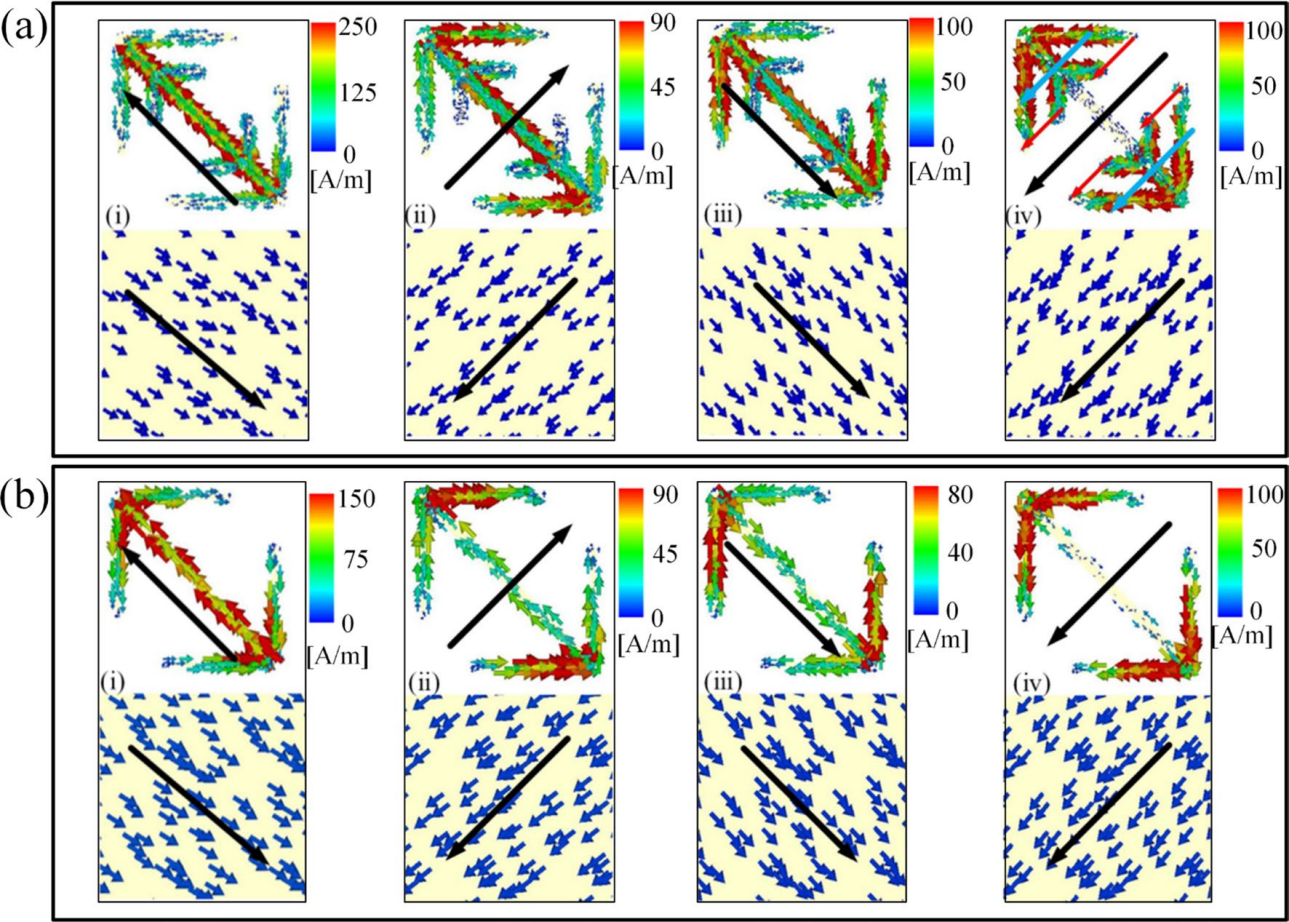
**(a)** Surface current distributions of unit 2 on the metallic parts of the metasurface unit cell and metallic ground sheet at first four resonance frequencies: (i)3.25 GHz, (ii) 4.81 GHz, (iii) 8.24 GHz, (iv) 11.69 GHz. **(b)** Surface current distributions of unit 1 on the metallic parts of the metasurface unit cell and metallic ground sheet at four resonance frequencies: (i) 3.08 GHz, (ii) 4.46 GHz, (iii) 7.34 GHz, (iv) 11.07 GHz.

In order to judge what type of each resonance, it is better to analyze surface current distributions. Therefore, we monitor the surface currents at each eigen-frequency as shown in Fig. 4. The first four resonances are analyzed firstly in Fig. 4a. Meanwhile, the surface current distributions of unit 1 are given in Fig. 4b regarded as comparisons. At the end of this section, the fifth resonance will be discussed.

Magnetic resonances are generated by anti-parallel currents coupling between top metal pattern and the background sheet. Electric resonances are generated by parallel currents. Fano resonance can be introduced by the strong interaction between bright and dark modes. More intuitively, Fano resonances can be caused by the interaction between the electric dipoles^34,35^.

Aiming to make it more intuitive, the partial surface current is shown by the arrows painting in the Fig. 4. We can regard V-shaped resonator as evolvement from cut-wire resonator and the V-shaped resonator will function as an extended cut-wire resonator in the *v*-direction. Therefore, in Fig. 4a, the magnetic resonance is generated by anti-parallel surface currents induced by extended cut-wire resonator and the ground sheet for (i) and (ii). And the electric resonance is generated by parallel surface currents induced by extended cut-wire resonator and the ground sheet for (iii). In (iv), according to the vector synthesis principle, two red arrows can be combined into a blue one and two blues constitute one black. The overall direction of surface current is still down to the left, which means it is parallel surface currents induced along resonator and the ground sheet. Therefore, it is still an electric resonance.

Besides, we also simulate the surface current distributions of unit 1 shown in Fig. 4b aiming to figure out what causes the Fano resonance. As is indicated by the above analysis, (i) and (ii) are magnetic resonance while (iii) and (iv) are electric resonance. Compared with the Fig. 4a (i)–(iv), the four resonance types of the unit 1 are basically same as the first four resonance of unit 2. Hence, it is the two sub-branches resonators that cause the Fano resonance.

**Figure 5:**
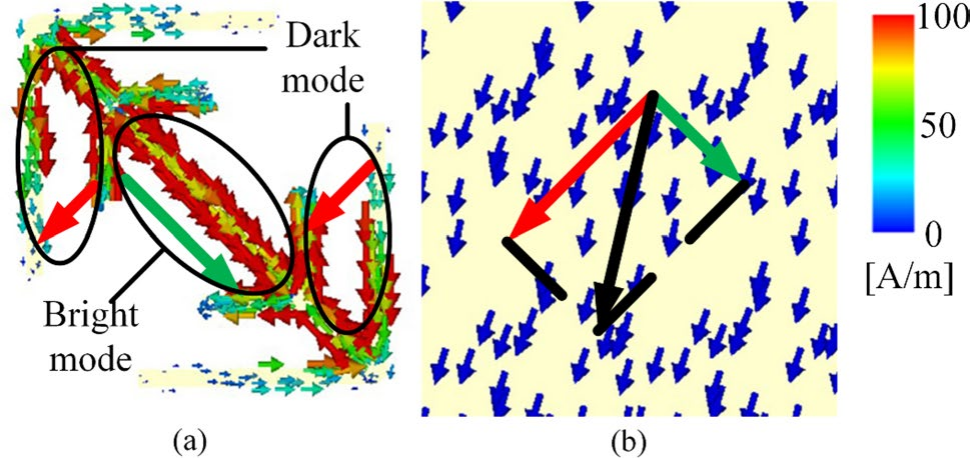
Surface current distributions of top metal pattern **(a)** and surface current distributions of bottom background sheet **(b)** at the fifth resonance frequency of 13.30 GHz.

Fano resonances suggest a good approximation of constructive and destructive interference of discrete states and continuum states, which are viewed as dark modes and bright modes^36,37^. From the Fig. 5a, the part that is circled in the middle can be seen as a “bright mode” for the parallel currents on both sides of the cut-wire. The other two circled parts can be regarded as “dark modes” because the opposite currents cancel each other out. The special structure of unit 2 leads to coexistence of both bright and dark modes at higher frequencies. Due to the strong interaction between the two modes, Fano resonance can be produced.

On the other hand, Fano resonances also can be intuitively explained by the coupled-mode theory of two classical Lorentzian oscillators with electromagnetic waves as driving force. In a case of two coupled oscillators, there are effectively two driving forces acting on the first oscillator, which are out of phase and cancel each other^38–40^. It can be seen from the Fig. 5b, there are both *u*- and *v*-direction of the surface current components in the ground sheet. In the *v*-direction of Fig. 5a, the surface current component is parallel to that in the middle part of unit 2 (green arrow), which will form an electrical resonance. In the *u*-direction, it is different for the surface currents between both sides of the cut-wire structure. From the color we can realize that the redder the color, the greater the current. Main surface currents are centralized on one side while there's almost nothing on the other side. Therefore, according to the vector synthesis principle, the main surface currents in *v*-direction are the two red arrows, which are parallel to *v*-direction surface current component in the ground sheet. Then, electrical resonances in the *u*-direction are formed. It is the interaction between the electric dipole and the quadrupole that causes Fano resonance with an asymmetrical spectrum. In conclusion, the first four resonances result in expansion of the operating frequency range because they are generated by electric and magnetic resonances. The fifth resonance is Fano resonance caused by interaction between dark modes and bright modes, which leads to further expansion of bandwidth.

**Figure 6:**
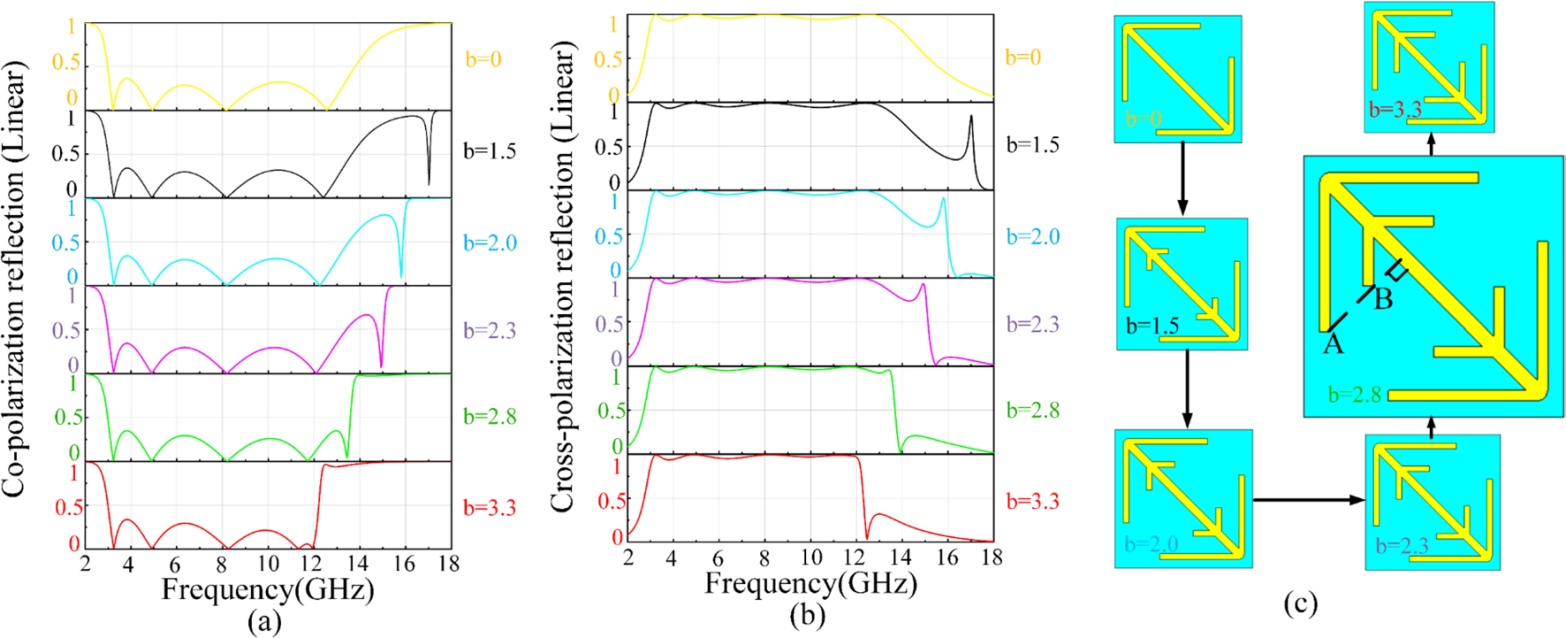
The linear co- **(a)** and cross-polarization **(b)** reflection when parameter *b* is 0, 1.5, 2.0, 2.3, 2.8, 3.3. And **(c)** is elevation view of structure which is changed with parameter *b*.

It is worth mentioning that the length of oblique V-shaped wires (parameter *b*) is a critical factor affecting the bandwidth and the performance of Fano resonance. As exhibited in Fig. 6a,b, we simulate the co- and cross-polarization reflection when parameter *b* is 0, 1.5, 2.0, 2.3, 2.8, 3.3. Compared *b* = 1.5 with *b* = 0 in Fig. 6a,b, we find that an obviously asymmetric spectral shape appears from 16 to 18 GHz and it is a typical characteristic of the Fano resonance. It is proofed again that the two V-shaped sub-branches resonators cause the Fano resonance. Furthermore, we find that the adjustment of parameter *b* can almost independently change the frequency of Fano resonance, and the resonant frequency will decrease with the increase of parameter* b* while have almost no effect on the first four resonances. Although the Fano resonance has a characteristic of narrow band, it still can be regarded as a method to further expand bandwidth on the basis of an ultra-wideband polarization conversion metasurface. Hence, in the principle of ensuring high efficiency while maximizing bandwidth, we successfully connect the narrow band of the Fano resonance with the wide band caused by the first four Lorentz resonances by adjusting parameter *b* to 2.8, as shown in the green lines of Fig. 6a,b. In Fig. 6b, the cross polarization of *b* = 2.8 does increase by a peak around 13.3 GHz compared with *b* = 0 and *b* = 1.5. Meanwhile, in the process of changing parameter *b* as shown in Fig. 6c, we find that the effect of Fano resonance will achieve best when the line between points A and B is approximately perpendicular to the cut-wire. This will provide a more intuitive strategy of the design in the future.

## Experiment results

**Figure 7:**
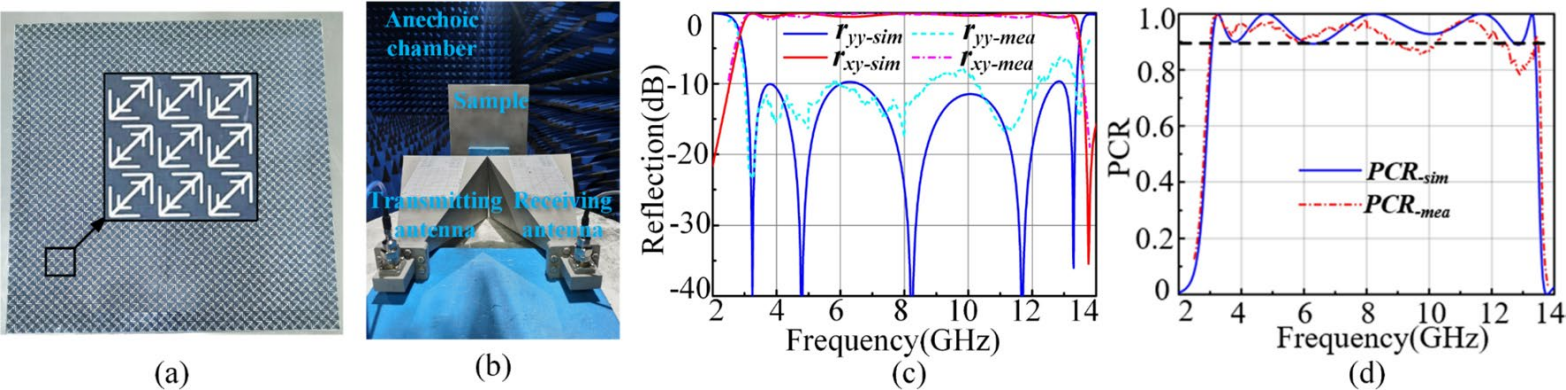
The fabricated sample **(a)** and the experimental setup **(b)** with measurements. The simulated and measured polarization reflection **(c)** versus frequency under normal incidence and the PCR **(d)** for the converter under a *y*-polarized incident wave.

To further verify the properties, we fabricate an area of 427 mm*427 mm square prototype of the polarization conversion metasurface as shown in Fig. 7a. The sample is measured using two horn antennas and the Agilent E8363B network analyzer, which is surrounded by absorbing materials to avoid unwanted reflections from the environment. The experimental setup with measurements is shown in Fig. 7b. In the process of experiment, one horn antenna is used as the transmitting antenna and the other is used for receiving antenna. We can obtain the co-polarization reflective coefficients by placing the both antenna on longer sides. The cross-polarization reflective coefficients can be obtained by placing one antenna on its longer side and placing the other antenna on its shorter side.

Both the simulated and measured results are presented in Fig. 7c. The simulated and measured PCR is also given in Fig. 7d. Compared with simulated results, we find that the measured co-polarization reflection is a little bit higher than that of simulation and the measured PCR is less than theoretical value. After further simulation and experimental verification, the little difference between measurement and simulation is caused by the distance between F4B layer and copper ground sheet. When the distance is greater than ${h}_{2}$, the last peak will be above theoretical value leading to the decrease of PCR. It is the hard-controllable thickness of foam and the interval between layers that lead to deviating from the theoretical value. On the other hand, the cross-polarization reflections are roughly the same as the theoretical value exhibiting a higher capacity of polarization conversion. In general, the experiment results are in agree with simulation results, which further verify that unit 2 has a wide and efficient bandwidth for both normally incident *x*- and *y*-polarized waves.

## Conclusion

The novelty in terms of mechanism of this paper is the introduction of Fano resonance to expand bandwidth because Lorentzian resonances are often used to expand bandwidth but Fano resonances are seldomly used. Besides, compared with *Chen *et al.^6^, the structure proposed this paper has a better performance. Ref.^6^ has a 1:4 bandwidth with PCR above 50%, while we have a 1:4.4 bandwidth with almost all PCR above 90%. Table 1 presents a comparison between the proposed reflective linear polarization converter and other reported ones operated in microwave frequency ranges. From Table 1, it can be observed that the proposed converter has an ultra-broadband and high-efficiency performance. In general, high-efficiency comes at the decrease of bandwidth. But we achieve high-efficiency and wideband simultaneously.

**Table 1:** Comparison with other single-layer wideband polarization converters.

References	Operation bandwidth (GHz)	Relative bandwidth	Efficiency (%)
Ref^6^. (2014)	6.2–24.3	1:4	50
Ref^21^. (2018)	6.67–17.1	1:2.6	90
Ref^33^.(2018)	5–15	1:3	50
Ref^32^.(2019)	4–8	1:2	90
Ref^22^.(2020)	8.85–18.85	1:2.13	95
Present study	3.01–13.4	1:4.4	90

Furthermore, the linear polarization conversion metasurface is a very basic work and if the foundation work is improved, any practical application of the foundation work will be expanded. For example, it can be used to achieve more radar cross section (RCS) reduction by combining with its 90°, 180° and 270° rotated ones to create the destructive interferences cancellation. Meanwhile, the proposed structure is a potential candidate for many polarization control devices due to its ultra-wideband and high efficiency performance or ultrasensitive sensors because the energy of Fano resonances is strongly confined which is suitable for switching devices, sensors, isolators, etc. This work also provides a notable avenue to metasurface bandwidth extension using Fano resonances and can also be extended to higher bands such as THz and infrared frequencies.
